# Editorial: The effect of COVID-19 on hematological disease diagnosis, management and outcomes

**DOI:** 10.3389/fmed.2023.1186934

**Published:** 2023-04-13

**Authors:** Mohamed Yassin, Ahmed Adel Elsabagh, Duygu Aydemir, Yasser Wali

**Affiliations:** ^1^Hematology Section, Medical Oncology, National Center for Cancer Care and Research (NCCCR), Hamad Medical Corporation (HMC), Doha, Qatar; ^2^College of Medicine, QU Health, Qatar University, Doha, Qatar; ^3^Department of Medical Biochemistry, School of Medicine, Koc University, Istanbul, Türkiye; ^4^Koç University Research Center for Translational Medicine (KUTTAM), Istanbul, Türkiye; ^5^College of Medicine and Health Science, Sultan Qaboos University, Muscat, Oman

**Keywords:** COVID-19, hematology, disease, outcome, comorbidity, complications

Lee, Chieng, Lau et al. studied the effects of coronavirus disease-2019 (COVID-19) on the clinical presentation, radiological findings, and outcomes of the infection in patients with hemoglobinopathies. They found that the possibility of severe COVID-19 infection in patients with hemoglobinopathies was higher than in the general population, with a percentage of 35.8% with severe infection compared to (11.1–19.1%) in the general population. The mortality rate was also higher than in the general public, with 6.9% mortality in patients with hemoglobinopathies compared to (2.2–5%) in the population. They also reported that hemoglobinopathy patients had higher mortality rates than patients with comorbid conditions such as chronic kidney diseases, but lower mortality rates than patients with HIV and malignancies. However, it was found that other comorbid conditions such as respiratory and cardiovascular diseases within these patients significantly increased mortality. It has been previously shown in several studies that comorbidities like hypertension could lead to a more aggressive course of COVID-19 infection and possible ICU admission (1). In addition, healthcare disparity and socioeconomic status in patients may have also led to worsened outcomes. Nevertheless, the review showed that patients with hemoglobinopathies suffer the same common COVID-19 symptoms occurring in the population. In addition, patients with hemoglobinopathies suffered from increased rates of vaso-occlusive crises secondary to hypoxia. This review and its reported numbers, however, included studies published during the earlier phases of the COVID-19 pandemic that included small sample sizes. Long-term effects were also not recorded. Finally, the radiological findings were descriptive and did not include definitive diagnoses and causes of these findings, which could be a limitation to this study as some radiological features can be found in both COVID-19 and some hemoglobinopathies like sickle cell disease (SCD). The review provides valuable insights into the management of COVID-19 in patients with hemoglobinopathies and underscores the need for further research in this (Lee, Chieng, Lau et al.).

Similarly, Martin et al. reviewed the clinical outcomes of children and adolescents with SCD who were infected with COVID-19. The study was conducted in a metropolitan tertiary pediatric hospital. The study concluded that these patients had a higher risk of conducting severe disease and more complications when compared to the general pediatric population. Nearly half (47%) of the patients with SCD and COVID-19 required hospitalization, and 5% were admitted to the ICU. Two out of the three patients admitted to the ICU were not on hydroxyurea. Vaso-occlusive crises, fever, and acute chest syndrome were the most common symptoms in hospitalized patients. No mortality was reported in the study. Compared to adults, pediatric patients with SCD may have had lower mortality due to lower incidences of end-organ damage and most patients were on disease-modifying therapy. It was shown that hydroxyurea treatment had a protective effect. It was suggested that pediatric patients with SCD should perhaps be prioritized in taking the COVID-19 vaccine as it may contribute to reducing the rate of complications and hospitalizations that these groups of patients are more prone to experience. The limitation of this study is that it is a retrospective analysis from a single hospital with a small sample size, so further data may be necessary to confirm their findings (Martin et al.).

Both Lee, Chieng, Abdul Jalal et al. and Marhaeni et al. studied the association between COVID-19 infection and ferritin levels. Lee, Chieng, Abdul Jalal et al. conducted a meta-analysis to study the relationship between serum ferritin levels and COVID-19 outcomes in patients with SCD. They found that even though elevated serum ferritin levels were commonly observed in sickle cell disease patients with COVID-19, they could not be used as a reliable predictor of severe disease or poor outcomes. Nevertheless, they found that ferritin levels cannot be used reliably to predict severity, ICU admission, or mortality in SCD patients. The limitations of the review include having a small number of eligible studies and limiting the generalizability of data. The authors suggest the need for further analysis and studies with larger sample sizes and better designs to confirm their findings (Lee, Chieng, Abdul Jalal et al.). Marhaeni et al., on the other hand, studied the levels of ferritin in COVID-19 infections in transfusion-dependent thalassemia (TDT) patients. However, a limitation to any study involving this population is that high ferritin levels cannot be certainly considered a result of COVID-19 infection as TDT patients normally present with elevated ferritin levels. This study included fourteen pediatric patients. They found that ferritin levels increased significantly in the infection period and decreased during the recovery period compared to baseline ferritin levels. It showed that even though serum ferritin levels were markedly and significantly increased in TDT patients after getting a COVID-19 infection, this rise in ferritin did not reflect the severity of the disease or symptoms. This is further proof that ferritin levels may not be reliable to use as a prognostic value in patients with hemoglobinopathies (Marhaeni et al.).

Sekizawa et al. published a case report about an 80-year-old Japanese woman who experienced a malignant marginal zone B-cell lymphoma after receiving the BNT162b2 mRNA COVID-19 vaccine. The characteristics upon the initial visit, which was 1 day after vaccine administration, showed signs of vaccine-related cervical lymphadenopathy, increasing the suspicion of malignancy. The radiological findings showed that the mRNA COVID-19 vaccine-related lymphadenopathy can be indistinguishable from neoplastic lymphadenopathies except for the absence of irregular margins. The study suggests that even though lymphadenopathy was common after mRNA vaccine administration, patients with COVID-19 vaccine-related lymphadenopathy should be regularly followed up and get comprehensive care. They also suggest close observation of lymph node enlargement which might occur later (up to 4–6 weeks) after vaccination and to avoid overlooking slowly progressing lymphadenopathies. Further studies are also necessary to test the causal relationship between COVID-19 vaccination and lymphoma progression (Sekizawa et al.).

Another case report published by Gogia et al. describes a case of Rosai-Dorfman-Destombes disease (RDD) that occurred in a 55-year-old woman following COVID-19 infection. This describes a case in which COVID-19 may have caused a histiocytic disease which could be due to immune dysregulation. However, further research is needed to confirm this hypothesis. Interestingly, this case also had the mRNA vaccination months before being infected and contracting the histiocytic infection. The patient was treated with corticosteroids and showed significant improvement in his symptoms (Gogia et al.).

Abuawwad et al. reviewed the existing literature on the association between ABO blood groups, Rh-factor, and COVID-19. The review concluded that individuals with blood group O had a lower chance of being infected with COVID-19 in addition to less chance of worsening. Individuals with blood group A on the other hand were found to be more prone to complications and getting infected. This, however, was not consistent across all studies. Some of the studies also suggested that individuals with Rh-positive blood may be at higher risk for severe COVID-19 disease, although evidence is conflicting. The authors suggest that these associations may be related to the interaction between the virus and the ABO antigens on red blood cells, although the underlying mechanisms are not yet clear. The article also notes that while the association between ABO blood groups, Rh-factor, and COVID-19 is of scientific interest, it should not be used as a basis for discrimination or stigmatization of individuals based on their blood type or Rh-factor status (Abuawwad et al.).

Some studies also investigated the hematological manifestations of COVID-19. This could be an important aspect to understand disease processes in COVID-19 infections. Some studies have previously shown, for instance, a protective effect of eosinophilia in patients with COVID-19 (2). Elemam et al. studied the morphologic and quantitative abnormalities in the peripheral blood counts of patients with (COVID-19) infections in the United Arab Emirates (UAE). The most common feature they found was the presence of atypical lymphocytes. Other commonly seen features were monocytes with cytoplasmic vacuoles and neutrophilic changes. They did not find significant changes in platelet counts. RBC changes like anisocytosis and hyperchromicity were found in about 50% of patients. Morphological changes in RBCs were also seen in many samples. They also tested the significance of the association between these changes and disease severity. However, significant differences were only seen with thrombocytosis and microcytic RBCs being significantly more common in stable patients compared to critical patients. They also showed that gender was associated with some specific abnormalities such as normochromic RBCs being associated with the male gender and a collateral decrease in hypochromic RBCs in males. The study had several limitations including the absence of any information regarding patient mortality as well as the absence of any healthy controls. Hence, further studies are needed to confirm these findings and to investigate the underlying mechanisms responsible for these hematological abnormalities in COVID-19 patients (Elemam et al.).

Aydemir and Ulusu performed a review of the available literature on the occurrence of thrombotic events after COVID-19 vaccination in patients with hematological disorders and hypercoagulable states. They found that these patients may be at increased risk of hypercoagulability after COVID-19 vaccination compared to the general healthy population. Thrombosis, portal vein thrombosis (PVT), immune thrombotic thrombocytopenia (ITT), deep vein thrombosis (DVT), vaccine-induced immune thrombocytopenia (VITT), and heparin-induced thrombocytopenia (HIT) are reported as vaccine-induced adverse effects in people with blood disorders. This led to the recommendation of administering anticoagulants following vaccination by the Expert Hematology Panel in March 2021. These findings were also supported by other studies that showed an increase in VTE prevalence in severe cases of COVID-19 infection (3). In conclusion, the study suggested closer monitoring during infection and after the administration of vaccines (Aydemir and Ulusu).

An article by Zomerdijk et al. investigates the factors associated with changes in healthy lifestyle behaviors among hematological cancer patients during the COVID-19 pandemic. The authors found that a considerable proportion of patients reported a decrease in physical activity and a decrease in the adoption of a healthy diet. The study highlights the importance of supporting healthy lifestyle behaviors among hematological cancer patients, particularly during the pandemic, to improve their overall health and wellbeing (Zomerdijk et al.).

An article by Böning et al. provides an update on the oxygen-carrying capacity and affinity in patients with COVID-19. The article discusses how COVID-19 affects the oxygen dissociation curve and can lead to hypoxemia, or low blood oxygen levels, which can contribute to respiratory failure and other complications. The authors also discuss potential treatment strategies to address hypoxemia in COVID-19 patients, including high-flow nasal cannula therapy and non-invasive positive pressure ventilation. They also note the importance of considering individual patient factors such as comorbidities and disease severity when choosing treatment strategies. A possible association was found between severity and the oxygen dissociation curve. To elaborate, they found that critical patients usually demonstrate a left shift of the curve which shows increased affinity. However, this feature was also associated with a good prognosis (Böning et al.).

## Author contributions

All authors listed have made a substantial, direct, and intellectual contribution to the work and approved it for publication.
